# Real Estate in the DNA Damage Response: Ubiquitin and SUMO Ligases Home in on DNA Double-Strand Breaks

**DOI:** 10.3389/fgene.2016.00058

**Published:** 2016-04-11

**Authors:** Nico P. Dantuma, Annika Pfeiffer

**Affiliations:** Department of Cell and Molecular Biology, Karolinska InstitutetStockholm, Sweden

**Keywords:** DNA damage, DNA double-strand breaks, SUMO, ubiquitin, chromatin

## Abstract

Ubiquitin and the ubiquitin-like modifier SUMO are intimately connected with the cellular response to various types of DNA damage. A striking feature is the local accumulation of these proteinaceous post-translational modifications in the direct vicinity to DNA double-strand breaks, which plays a critical role in the formation of ionizing radiation-induced foci. The functional significance of these modifications is the coordinated recruitment and removal of proteins involved in DNA damage signaling and repair in a timely manner. The central orchestrators of these processes are the ubiquitin and SUMO ligases that are responsible for accurately tagging a broad array of chromatin and chromatin-associated proteins thereby changing their behavior or destination. Despite many differences in the mode of action of these enzymes, they share some striking features that are of direct relevance for their function in the DNA damage response. In this review, we outline the molecular mechanisms that are responsible for the recruitment of ubiquitin and SUMO ligases and discuss the importance of chromatin proximity in this process.

## Introduction

The cellular response to compromised genome integrity is a vital process that is tightly regulated by a number of post-translational modifications (PTMs) that dictate the course of action at the sites of DNA damage. While ensuring that proper action will be taken to eliminate the threat, these regulatory circuits at the same time avoid unnecessary and potentially hazardous activation of DNA repair pathways. In this review, we will focus on ubiquitin and the small ubiquitin-like modifiers (SUMO)-1, -2, and -3, which are central players in this process, where they in tight conjunction with other PTMs – most notably phosphor-modifications but also another ubiquitin-like protein modifier Nedd8 – activate signaling cascades and coordinate mobilization of the proper DNA repair machinery (Bekker-Jensen and Mailand, 2011; Jackson and Durocher, 2013). Rather than providing a complete overview of the rapidly expanding number of ligases that are involved in this process, we will focus on a limited set of ligases that illustrates the importance of proximity to DNA lesions in DNA damage-induced ubiquitylation and SUMOylation.

Modification of chromatin and chromatin-associated proteins by these PTMs in response to DNA double-strand breaks (DSBs) results in the formation of the characteristic ionizing radiation-induced foci (IRIF) that mark the sites of DNA damage (Lukas et al., 2011). In contrast to phosphorylation at IRIF, which is primarily facilitated by the PI3K-like kinase ATM with the variant histone H2AX being the predominant target (Shiloh, 2003), decoration of the chromatin with ubiquitin and SUMO is attributed to several enzymes that differ in their specificity for substrates at the chromatin (Bekker-Jensen and Mailand, 2011; Jackson and Durocher, 2013). Despite the many differences between the ubiquitin and SUMO ligases involved in the DNA damage response, they share a number of characteristics such as the critical role of chromatin recruitment for their functionality and their tendency to target multiple substrates at the DSBs.

## Regulation by Proximity

**FIGURE 1:**
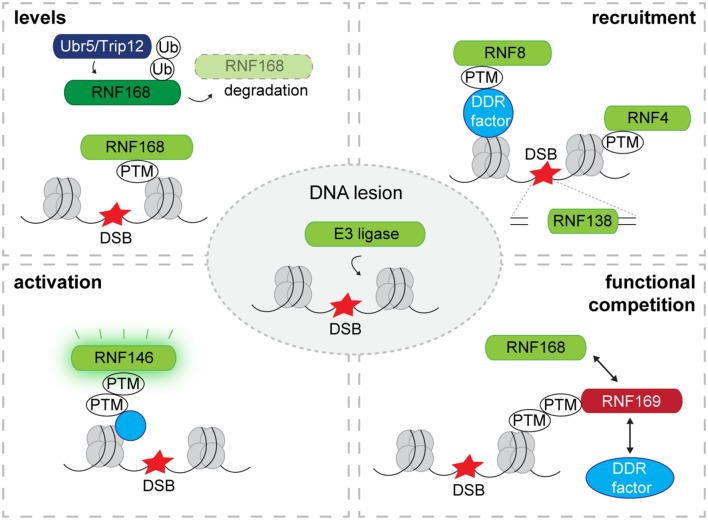
**Recruitment and regulation of E3 ligases at DNA lesions.** Levels: E3 ligases are targeted by other E3 ubiquitin ligases for proteasomal degradation. Proteasomal degradation of E3 ligases limits the quantity of E3 ligases that translocate to DNA lesions as has been observed for RNF168 which is targeted for degradation by TRIP12 and UBR5. Recruitment: The recruitment of E3 ligases to DSBs is often mediated by PTMs that are attached to the chromatin or chromatin-associated proteins. Some E3 ligases are recruited by directly binding to free DNA ends exposed at DSBs. Activation: While most E3 ligases in complex with their E2 possess constitutive enzymatic activity, a PTM can activate E3 ligase activity as has been observed for the PAR-dependent ubiquitin ligase RNF146. Competition: Competitors negatively regulate the recruitment of E3 ligases by binding to PTMs that facilitate the binding of E3 ligases as has been observed for RNF168 and its competitor RNF169. DSB, DNA double-strand break; Ub, ubiquitin; PTM, post-translational modification; DDR, DNA damage response.

An important mechanistic difference between the DSB-induced phosphorylation and ubiquitin/SUMO response at IRIF lies in the way their activity is regulated. While the activity of ATM is kept dormant in undamaged cells only to be unleashed upon the detection of DSBs (Bakkenist and Kastan, 2003), most of the enzymes that are responsible for conjugation of ubiquitin and SUMO at sites of DSBs lack direct activation mechanisms. Despite the fact that additional regulatory mechanisms may be in play, a general concept appears to be the DNA damage-induced translocation of ligases to the DSBs as a primary determinant for directing the activity of these enzymes toward chromatin and chromatin-associated proteins (**Figure 1**).

**FIGURE 2:**
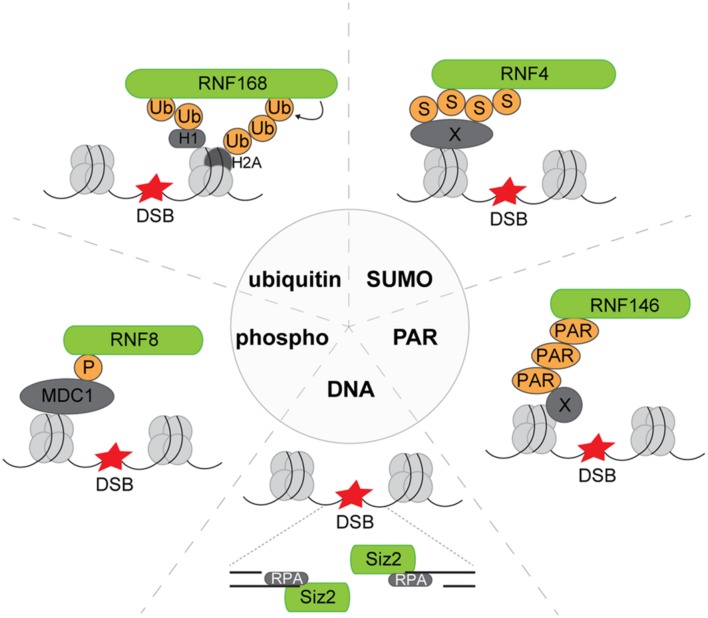
**Recruitment modes of exemplary E3 ligases to DNA lesions.** Phosphor: phosphorylated MDC1 serves as the recruitment platform for RNF8 to DNA lesions. Ubiquitin: RNF168 binds to both ubiquitylated linker histone H1 and ubiquitylated core histone H2A. SUMO: RNF4 harbors SUMO-interacting motifs by which it can bind to SUMOylated substrates, e.g., MDC1 at DNA lesions. PAR: The E3 ligase RNF146 translocates to DNA breaks and is activated by binding to PARylated substrates. DNA: By binding to ssDNA and RPA, the yeast SUMO ligase Siz2 is recruited to DNA lesions. DSB, DNA double-strand break; Ub, ubiquitin; S, SUMO, small ubiquitin-like modifier; PAR, poly(ADP-ribose); P, phosphate group; X, variable or unspecified protein.

Various PTMs and also the exposure of single-stranded DNA (ssDNA) triggers the accrual of ubiquitin and SUMO ligases (**Figure 2**). RNF8 and RNF168 are two RING ubiquitin ligases that play an important role in the DSB-induced ubiquitylation response and act downstream of the ATM-dependent phosphorylation triggered by DNA damage (Huen et al., 2007; Kolas et al., 2007; Mailand et al., 2007; Doil et al., 2009; Stewart et al., 2009). RNF8 interacts with its FHA domain to ATM-phosphorylated MDC1, which in turn binds ATM-phosphorylated variant histone H2AX (γH2AX), a hallmark of IRIF (Huen et al., 2007; Kolas et al., 2007; Mailand et al., 2007). A dual recruitment mechanism is involved in accrual of RNF168, which has one binding module that facilitates interaction with linker histone H1 modified with non-proteolytic lysine 63 (K63)-linked ubiquitin chains (Thorslund et al., 2015) and a second binding module that recruits it to the core histone H2A/H2AX ubiquitylated at lysine residues K13/K15 (Panier et al., 2012). The latter modification is generated by RNF168 and provides a positive feedback loop that amplifies RNF168-mediated ubiquitylation (Mattiroli et al., 2012). The RNF8/RNF168-mediated ubiquitylation response results in the recruitment of proteins involved in the repair of DSBs, such as BRCA1 and 53BP1, to the chromatin at sites of DNA damage.

Artificial tethering of these two ubiquitin ligases to the chromatin is sufficient to locally reconstitute the DNA damage response to a large extent without inflicting actual DSBs (Acs et al., 2011; Luijsterburg et al., 2012). Interestingly, sequestration of RNF8 at chromatin resulted in the formation of foci that displayed many of the hallmarks observed at IRIF such as ubiquitylation of histone H2A, formation of K63-linked ubiquitin chains and recruitment of RNF168 and BRCA1 (Luijsterburg et al., 2012). While tethering of RNF168 did not result in accrual of RNF8, consistent with the notion that it acts downstream of RNF8, it also gave rise to H2A ubiquitylation and BRCA1 recruitment (Luijsterburg et al., 2012). Thus the mode of action of these two ubiquitin ligases illustrates that chromatin retention plays an important role in their regulation. It is tempting to speculate that the fact that they operate downstream of ATM and hence rely on activation of ATM provides sufficient safety measures to prevent random erroneous activation of the pathway at chromatin. Moreover, their constitutive activity may have advantages in the sense that it may allow these proteins to have other functions in the absence of DNA damage as has been documented for RNF8 (Takano et al., 2004).

Amplification of the ubiquitylation response at sites of DNA damage by RNF168 is essential for a robust DNA damage response and is hence tightly regulated at various levels (**Figure 1**). Two ubiquitin ligases, UBR5 and TRIP12, target RNF168 for proteasomal degradation and depletion of these ligases results in supraphysiological steady-state levels of RNF168 giving rise to superfluous activation of the ubiquitylation response at DSBs (Gudjonsson et al., 2012).

In addition, chromatin accrual of RNF168 is kept under control by its paralog RNF169 (Chen et al., 2012; Poulsen et al., 2012), which also binds to RNF168-generated ubiquitin chains but does not amplify the signal (Panier et al., 2012; **Figure 1**). Also activation of the DNA damage response by herpes simplex virus type 1 is prevented by the viral ubiquitin ligase ICP0 targeting RNF8 and RNF168 (Lilley et al., 2010). It is striking that these regulatory mechanisms target the steady-state levels and chromatin accrual of RNF168 and not its activity underscoring the importance of localization of this ubiquitin ligase in DNA damage signaling.

An exceptional case is the poly(ADP-ribose) (PAR)-dependent ubiquitin ligase RNF146, also known as Iduna, since DNA damage-induced PARylation not only induces its translocation but also releases its ubiquitin ligase activity (Kang et al., 2011; **Figure 1**). RNF146 selectively interacts with PARylated proteins at DSBs resulting in their ubiquitylation. Although its activity is not confined to PARylated proteins at DSBs (Zhang et al., 2011), the PAR-dependent recruitment of RNF146 is important for efficient DNA repair. Structural analysis revealed that interaction between PAR and the WWE domain of RNF146 switches its RING domain into an active state that promotes conjugation of ubiquitin to PARylated proteins (DaRosa et al., 2015). Thus, RNF146 is kept in a dormant state only to be activated upon interaction with PARylated substrates.

In contrast to the large number of ubiquitin ligases in metazoan cells, SUMO ligation is mediated by a selective set of dedicated enzymes. In particular SUMO ligases belonging to the PIAS family – Siz2 in yeast and PIAS1-4 in mammalian cells – have been implicated in the cellular response to DSBs. Also these enzymes modulate substrates at the chromatin as a direct consequence of their DNA damage-induced translocation to breaks (**Figure 1**). For yeast Siz2 it has been shown that once recruited it SUMOylates chromatin-associated proteins at DNA damage in a rather promiscuous fashion, a process that has been referred to as group modification (Psakhye and Jentsch, 2012). Strikingly, artificial tethering of proteins to the chromatin is sufficient to turn them into substrates for DNA damage-recruited Siz2 underscoring its ability to modify proteins primarily based on their proximity (Psakhye and Jentsch, 2012). Since the individual contribution of the SUMO modifications is limited while at the same time the presence of an active SUMOylation response is critical for homologous recombination, it has been proposed that the SUMO modifications may provide a “glue” that stabilizes local interactions by binding to SUMO-interacting motifs (SIMs), which are commonly found in DNA repair proteins (Jentsch and Psakhye, 2013). It is important though to mention that modification of specific substrates can also be highly relevant as has been shown for PCNA, which is SUMOylated at a specific lysine residue by Siz1, dictating the preferred mechanism for dealing with lesions that block replication forks (Mailand et al., 2013). DSBs in mammalian cells recruit the SUMO ligases PIAS1 and PIAS4 where they modify BRCA1, 53BP1 and other substrates with SUMO1 and SUMO2/3 conjugates (Galanty et al., 2009; Morris et al., 2009). The PIAS1/4-facilitated SUMOylation is critical for a functional DNA damage response and impediment of this process compromises recruitment of RNF168, 53BP1, and BRCA1 (Galanty et al., 2009; Morris et al., 2009). Although the general underlying molecular mechanism for the critical role of SUMO in DNA damage-induced ubiquitylation remains elusive, it has been shown that the ubiquitin ligase activity of the BRCA1/BARD1 complex is enhanced by SUMOylation, which may in part explain its stimulatory effect (Morris et al., 2009). It is not known whether similar group modifications are involved in this process but it is noteworthy that both in yeast and mammalian cells the role of SUMOylation is complex and can stimulate recruitment, retention or extraction depending on the nature of substrate and the context of the modification.

## Tracing DNA Lesions

The central role of the recruitment of ubiquitin/SUMO ligases in activation of DNA repair pathways also implies that their translocation to DSBs has to be tightly regulated. Notably, while lack of activation of DNA repair mechanisms or DNA damage signaling cascades in the presence of DSBs is dangerous for cells, inappropriate or superfluous activation of these systems form an equally serious threat. It is interesting that some of the ubiquitin ligases that are implicated in this process use analogous mechanisms for their recruitment and combine motifs that bind to specific DNA damage-induced PTMs with domains that interact with chromatin ensuring that these modifications will only be recognized as valid signals in the context of chromatin.

Proper accrual is of particular importance for the RNF168 ubiquitin ligase which is responsible for the amplification of the DNA damage-induced ubiquitylation response initiated by RNF8. While RNF168 is essential for recruitment of 53BP1 and BRCA1 and the actual repair of DSBs, excessive levels of RNF168 also compromise DNA repair (Gudjonsson et al., 2012). RNF168 specifically ubiquitylates histone H2A(X) in the context of the nucleosome by interacting through a basic region within its RING domain with an acidic patch that is present at the interface of the H2A/H2B dimer (Leung et al., 2014; Mattiroli et al., 2014). Binding of RNF168 to the nucleosome allows its cognate ubiquitin conjugase to transfer the ubiquitin to the target lysine residues within H2A(X). However, this direct interaction with the nucleosome is not sufficient for establishing chromatin retention since RNF168 has to selectively interact with ubiquitylated linker histone H1 (Thorslund et al., 2015) or the H2AK13,15ub mark (Mattiroli et al., 2012). Notably, RNF168 contains two recognition modules both involving motif-interacting with ubiquitin (MIU) domains that are specific for these modifications (Panier et al., 2012). Interestingly, its paralog RNF169, which suppresses DNA damage-induced ubiquitylation (Chen et al., 2012; Poulsen et al., 2012), only contains the module that facilitates interaction with the RNF168-generated H2AK13,15ub mark allowing it to inhibit the amplification of the signal by tempering with the initial activating response (Panier et al., 2012).

It is noteworthy that the BMI1/RING1b ligase, which is part of the polycomb recessive complex 1 (PRC1) that facilitates the canonical ubiquitylation of histone H2A at residue K119 (Sparmann and van Lohuizen, 2006), employs the same acidic patch to faithfully interact with the nucleosome (Leung et al., 2014; McGinty et al., 2014). Although it had been proposed that the BMI1/RING1b ligase interacts with nucleosomal DNA in a sequence-independent manner (Bentley et al., 2011), structural analysis showed that its cognate E2 UbcH5 facilitates this interaction (McGinty et al., 2014). Importantly, this ubiquitin ligase complex has also been linked to the DNA damage response, both at DSBs and UV lesions, where it monoubiquitylates histone H2AX and promotes the DNA damage response (Ismail et al., 2010). PRC1 accumulates at DSBs by a mechanism that is different from its well-established chromatin retention by PRC2-generated H3K27me^3^ and does not require DNA damage-induced γH2AX. PRC1 is also required for DNA damage-induced silencing at DSBs but this activity requires the presence of PRC2 suggesting that it is more similar to the canonical role of these complexes in suppression of transcription (Kakarougkas et al., 2014).

RNF4 is a SUMO-targeted ubiquitin ligase (STUbL) that selectively ubiquitylates proteins that have been modified by chains consisting of the highly related SUMO2 and SUMO3 modifiers, in particular under conditions of proteotoxic or genotoxic stress (Kosoy et al., 2007; Sun et al., 2007). In response to DSBs, RNF4 translocates to sites of DNA damage by interacting with its SIMs with chromatin-associated proteins that are subject to DNA damage-induced SUMOylation (Galanty et al., 2012; Luo et al., 2012; Yin et al., 2012). RNF4-mediated ubiquitylation of MDC1 and RPA results in removal of these proteins from DSBs and plays an important regulatory role. In addition to its interaction with the SUMO conjugates, the RING domain of RNF4 contains a nucleosome-interacting motif that is structurally related to the motifs in RNF168 and RING1b and which is required for targeting RNF4 to chromatin (Groocock et al., 2014). Although the nucleosome-interacting motif binds DNA, as in the case for RING1b, it is not clear whether DNA binding and/or histone interaction are responsible for its nucleosome targeting activity (Groocock et al., 2014).

Another recruitment mechanism is employed by the ubiquitin ligase RNF138, which stimulates repair of DSBs by homologous recombination. This ubiquitin ligase contains three zinc finger (ZnF) motifs that specifically interact with ssDNA overhangs at lesions (Ismail et al., 2015; Schmidt et al., 2015). Accordingly, RNF138 acts downstream of the Mre11 nuclease that is responsible for the generation of ssDNA at DSBs. RNF138-mediated ubiquitylation fulfills a dual role at DSBs since it facilitates the removal of the Ku70–Ku80 heterodimer (Ismail et al., 2015) and stimulates the recruitment of CtIP resulting in resection of DNA ends (Schmidt et al., 2015) and repair of the lesions by homologous recombination. Thus, unlike the above mentioned ubiquitin ligases, RNF138 localizes to the actual break and not to the chromatin in proximity of the DSBs and uses a dedicated recruitment motif to accomplish this. Also RNF111, which modifies histone H4 with chains of the ubiquitin-like modifier Nedd8, interacts with naked DNA and it has been proposed that this may serve to secure its recruitment to DSBs (Ma et al., 2013). Confusingly, the same ubiquitin ligase has also been reported to localize to UV damage in a SUMO-targeted fashion where it modifies its target XPC with ubiquitin chains instead of Nedd8 (Poulsen et al., 2013), resulting in chromatin extraction of XPC raising questions both about RNF111’s mechanism for accrual and mode of action (van Cuijk et al., 2015).

The generation of ssDNA is also important for the recruitment of the budding yeast SUMO ligase Siz2 to DSBs. Siz2 belongs to the family of PIAS ligases which have been found to be involved in the DNA damage response not only in yeast but also in human cells, in particular PIAS1 and PIAS4. Originally it was proposed that the conserved SAP domain in these SUMO ligases facilitates recruitment by binding to ssDNA which triggers a wave of early SUMOylation at DSBs (Psakhye and Jentsch, 2012). However, a recent study revealed that while the ssDNA is critical for translocation of Siz2, it does so by binding the ssDNA-binding complex RPA (Chung and Zhao, 2015). Siz2 interacts with this trimeric complex that coats ssDNA resulting in SUMOylation of RPA and other chromatin-associated targets. Also the PIAS1 and PIAS4 SUMO ligases interact with the same RPA subunit (Chung and Zhao, 2015) and accrual has been shown to be dependent on their N-terminal SAP domains (Galanty et al., 2009), suggesting that similar recruitment mechanisms may be in play in human cells. SUMOylation of RPA followed by ubiquitylation catalyzed by STUbLs results in chromatin eviction of these proteins and plays a critical role in regulating the repair of DSBs (Galanty et al., 2012).

## Concluding Remarks

The detailed insights in the recruitment mechanisms that regulate chromatin association of DNA damage ubiquitin and SUMO ligases and the important role of proximity in DNA damage-induced protein modifications stands in sharp contrast to our modest understanding of how these PTMs regulate the fate of the modified proteins. Importantly, ubiquitylation and SUMOylation have been shown to be stimulators of protein recruitment, retention and extraction, supposing opposite actions that are hard to reconcile in one mechanistic paradigm, raising questions of what determines the final biological outcome of these modifications. For example, RNF8-mediated ubiquitylation forms the docking platform for critical DNA repair proteins (Huen et al., 2007; Kolas et al., 2007; Mailand et al., 2007), while at the same time it has been shown to promote ubiquitin-dependent chromatin extraction of proteins (Acs et al., 2011; Meerang et al., 2011; Feng and Chen, 2012; Mallette et al., 2012). Also the STUbL RNF4 has been shown to select SUMOylated chromatin-associated proteins for eviction (Galanty et al., 2012; Luo et al., 2012; Yin et al., 2012) but has also been implicated in the recruitment of proteins to DSBs (Hendriks et al., 2015). The picture is further complicated by the notion that SUMO modifications can by themselves target proteins for extraction (Bergink et al., 2013), whereas at the same time SUMO group modification has been proposed to play a general role in stabilizing chromatin association of proteins (Psakhye and Jentsch, 2012). Decrypting the ubiquitin/SUMO code in the DNA damage response will be a major challenge for the future and may shed light not only on the molecular mechanisms that dictate the behavior of proteins at DNA damage but also other processes that have the chromatin environment as their central stage, such as transcription and replication.

## Author Contributions

All authors listed, have made substantial, direct and intellectual contributions to the work, and approved it for publication.

## Conflict of Interest Statement

The authors declare that the research was conducted in the absence of any commercial or financial relationships that could be construed as a potential conflict of interest.

## Abbreviations

DSB: DNA double-strand break
IRIF: ionizing radiation-induced foci
MIU: motif interacting with ubiquitin
PAR: poly(ADP-ribose)
PTM: post-translational modification
PRC: polycomb recessive complex
SIM: SUMO-interacting motif
STUbL: SUMO-targeted ubiquitin ligase
ZnF: zinc finger.
